# Treatment of Operating Theatre Walls

**Published:** 1906-10-27

**Authors:** E. A. Attwood

**Affiliations:** Secretary. London Homœopathic Hospital, Great Ormond Street


### Editor's Letter-Box.

[Our Correspondents are reminded that prolixity is a great bar to publication, and that brevity of style and conciseness of statement greatly facilitate early insertion.]

### TREATMENT OF OPERATING THEATRE WALLS.

Sir,—I notice in your issue of October 20th you recommend a good foundation of Keen's cement, with several coats over it of enamel paint, as the best arrangement for treating the walls of operating theatres. The walls of the operating theatre of this hospital are covered with Keen's cement, and have just been coated with "Ripolin" enamel, which gives a perfectly satisfactory and clean surface to the walls, and meets all aseptic requirements.

I shall be pleased to show the theatre to anyone interested upon application to

Yours faithfully,
E. A. Attwood, Secretary.

London Homœopathic Hospital,
Great Ormond Street, October 19th, 1906.

